# Detection of canine vector-borne diseases in eastern Poland by ELISA and PCR

**DOI:** 10.1007/s00436-015-4832-1

**Published:** 2015-11-19

**Authors:** Beata Dzięgiel, Łukasz Adaszek, Alfonso Carbonero, Paweł Łyp, Mateusz Winiarczyk, Piotr Dębiak, Stanisław Winiarczyk

**Affiliations:** Department of Epizootiology and Infectious Diseases, Faculty of Veterinary Medicine, University of Life Sciences in Lublin, 30 Głęboka St., 20-612 Lublin, Poland; Departamentoi de Sanidad Animal, Facultad de Veterinaria, UCO, Campus Universitarios de Rabanales|, 14071 Córdoba, Spain; Department of Vitreoretinal Surgery, Medical University of Lublin, Chmielna 1, 20-079 Lublin, Poland; Department and Clinic of Animal Surgery, Laboratory of Radiology and Ultrasonography, Lublin, Poland

**Keywords:** Vector-borne disease, *Anaplasma phagocytophilum*, *Borrelia burgdorferi*, *Ehrlichia canis*, Dogs, Poland

## Abstract

The aim of the study was to establish the prevalence of *Ehrlichia canis*, *Anaplasma phagocytophilum* and *Borrelia burgdorferi* in dogs in eastern Poland and to determine the factors associated with exposure (seroposity) or infection (PCR). Anti-*A. phagocytophilum*, anti-*B. burgdorferi* and anti-*E. canis* antibodies were determined in 400 dogs, using the SNAP 4Dx ® test (IDEXX Laboratories). In addition, PCRs were performed for the detection of *E. canis*, *A. phagocytophilum* and *B. burgdorferi* DNA. In reference to the risk factor analysis, a regression logistic model was determined for each aetiological agent. The overall seroprevalence was highest for *B. burgdorferi* (11.0 %), followed by *A. phagocytophilum* (8.0 %) and *E. canis* (1.5 %). Eleven healthy dogs were found to be infected with *A. phagocytophilum*, as determined by PCR, while the remainder were seronegative. For *B. burgdorferi*, the DNA of the spirochetes was detected in the blood of 20 dogs, while the presence of anti-*B. burgdorferi* IgG was detected in the sera of ten of these. For *E. canis*, none of the dogs tested positive by PCR. Tick control was included as a protective factor for *A. phagocytophilum* and *B. burgdorferi*, while the origin (rural) was included as a risk factor for *B. burgdorferi* and *A. phagocytophilum* infection. In addition, breed (pure) was a risk factor for *B. burgdorferi* infection, and sex (female) was a risk factor for *E. canis.*

## Introduction

The term canine vector-borne diseases (CVBD) includes a wide variety of diseases of infectious or parasitic aetiology whose agents are transmitted by ectoparasites such as ticks, fleas and mosquitoes (Otranto et al. 2009a). Control of these infectious agents is important because some are responsible for serious diseases in humans (e.g. *Anaplasma phagocytophilum* and *Borrelia burgdorferi*). However, their control can be a highly complex process since they show a wide geographical distribution while the clinical signs in infected dogs may vary significantly (Otranto et al. 2009b; Day 2011).

CVBD may show no specific clinical signs or clinical-pathological abnormalities and may even present a varied clinical picture. This makes the diagnosis of a CVBD extremely complex. Animals with subclinical infections pose as increased risk of infection (Billeter et al. 2008; Perez et al. 2011).

Infections with *A. phagocytophilum*, the causative agent of human granulocytic anaplasmosis, have been increasingly diagnosed in both companion and farm animals in Poland (Adaszek et al. 2009; Zygner et al. 2009; Dzięgiel et al. 2013). This pathogen has also been found in the blood of game animals (Adaszek et al. 2012; Dzięgiel et al. 2015). *A. phagocytophilum* usually causes an acute infection in dogs, characterized by fever and thrombocytopenia, although subclinical infections have been reported. This pathogen was recently observed in 10.3 % of *Ixodes ricinus* ticks studied from the eastern part of Poland (Dzięgiel et al. 2014).

Lyme disease is an infectious disease caused by the spirochetes *B. burgdorferi sensu lato* complex, transmitted by ticks of the genus *Ixodes* (Pantchev et al. 2015). *B. burgdorferi* affects a wide range of hosts, mainly humans and dogs. In dogs, Lyme disease can produce chronic weakness with nonspecific clinical signs (fever, muscle and joint pain). Although some dogs show clinical signs, mostly the infection is subclinical (Pantchev et al. 2015). In Poland, dogs showing seropositive for *B. burgdorferi* have been detected in the Lubelskie Voivodeship (6.3 %) (Adaszek et al. 2008).

*Ehrlichia canis* is the causative agent of canine monocytic ehrlichiosis and is transmitted by *Rhipicephalus sanguineus*. Three clinicopathologic stages of ehrlichiosis have been recognized in dogs: an acute stage where dogs show variable clinical signs (such as lethargy, fever, lymphadenomegaly and epistaxis) and thrombocytopenia; a subacute phase characterized by hyperglobulinemia, thrombocytopenia and anaemia; and a chronic stage where the dogs may give variable clinicopathologic findings (lethargy, thrombocytopenia, pancytopenia) while remaining seropositive (Pérez Vera et al. 2014).

The present study was designed to establish the serological and molecular prevalence (*E. canis*, *A. phagocytophilum*, *B. burgdorferi*) of selected tick-borne diseases in dogs in eastern Poland and to determine the epidemiological factors associated with exposure and/or infection.

## Material and methods

### Animals and sampling

In the period 2011–2014, serum samples from 400 healthy dogs randomly sampled from 23 clinics from eastern Poland (totally 400 animals, from four voivodeships—100 hundred dogs from each voivodeship) were examined for anti-*B. burgdorferi*, anti-*A. phagocytophilum* and anti-*E. canis* antibodies. None of the dogs left the Polish territory. The dogs were referred to the clinics for prophylaxis reasons (vaccination, prophylaxis against ecto- and endoparasites). Serum and whole blood samples were taken from all animals for serological and molecular study to test for these three pathogens. The samples were collected by veterinary practitioners upon request.

The study was reviewed and approved by the Ethics Committee of the University of Life Sciences in Lublin, No. 80/2010.

Epidemiological data from all the dogs were recorded by means of a questionnaire, completed for each animal and containing the following information:

- Region: Dogs from four voivodeships (the highest level administrative subdivision of Poland, corresponding to a “province” in many other countries) were examined. The study comprised dogs from four voivodeships located in eastern Poland (*Lubelskie*, *Podlaskie*, *Mazowieckie* and *Podkarpackie*). The characteristics of the regions are presented by Gorzelak et al (2006).
- Breed and sex: the sample included dogs from many breeds (mixed breed dogs, *n* = 280; German shepherd, *n* = 34; Bernese mountain, *n* = 22; Rottweiler, *n* = 11; Cocker spaniel, *n* = 10; Siberian husky, *n* = 10; American staffordshire terrier, *n* = 10; Welsh corgi cardigan, *n* = 5; Fox terrier, *n* = 4; Schnauzer, *n* = 4; Boxer, *n* = 3; Dachshund, *n* = 3; Beagle, *n* = 2; and Pointer, *n* = 2), including both male (*n* = 291) and female (*n* = 109) animals. In consequence, it was decided to classify by pure or mixed breeds.
- Origin: the animals were classified by whether they come from rural (*n* = 157) or urban (*n* = 243) areas.
- Use of tick- or vector-borne disease control measures (prophylaxis against ectoparasites in a form of spray, collar or spot on) (*n* = 306).
- Age: the animals were classified as young animals (under 1 year old) (*n* = 115) or adult animals (1 year or older) (*n* = 285).

### Serological testing

Serum samples were tested using a qualitative dot-ELISA SNAP 4Dx ® (IDEXX Laboratories). It detects *Dirofilaria immitis* antigen, antibodies against *B. burgdorferi* s.l. and antibodies against four intracellular bacteria of the order *Rickettsiales*: *A. phagocytophilum/Anaplasma platys* and *E. canis/Ehrlichia ewingii*. Because so far on Polish territory *A. platys* and *E. ewingii* infections were not diagnosed, and bearing in mind the fact that the animals used in the study had never left the country, the antibodies detected in the serum of dogs for *A. phagocytophilum*/*A. platys* were considered as anti-*A. phagocytophilum* and for *E. canis/E. ewingii* were considered as anti-*E. canis*

### DNA extraction and PCR amplification

DNA extractions for molecular tests were performed using the DNA Blood kit (A&A Biotechnology Gdańsk, Poland). The extracted DNA was subjected to PCR.

PCR was performed according to the method described by Skotarczak et al. (2005) and Adaszek et al. (2009) with the primers SC1 (5′-GCT GTC AGT GCG TCT TAA-3′) and SC2 (5′-CTT AGC TGC TGC CTC CGT A-3′), used to amplify the 16S rRNA gene fragment of *B. burgdorferi* s.l., and EHR 521 (5′-TGT AGG CGG TTC GGT AAG TTA AAG-3′) and EHR 747 (5′-GCA CTC ATC GTT TAC AGC GTG-3′), enabling amplification of the 16S rRNA gene fragment of *Anaplasma*/*Ehrlichia* spp. The expected product sizes were approximately 300 bp for *B. burgdorferi* s.l. and 247 bp for *Anaplasma*/*Ehrlichia* spp. The final identification of the *Ehrlichia/Anaplasma* spp. was done based on results of sequencing of the obtained PCR products. For use as a positive control, the DNA of *B. burgdorferi* s.l. and DNA of *A. phagocytophilum* were obtained from the National Reference Center for Borrelia of the Max von Pettenkofer Institute. Pure water was used as negative control. PCR amplification was performed using a programmable thermocycler (Biometra, Goettingen, Germany).

The size of each PCR product was analysed by electrophoresis in a 1.5 % agarose gel stained with ethidium bromide. The products were purified using QIAquick spin columns (Qiagen) and eluted in 50 μl of Tris10 mM, pH 7.6. DNA sequencing was performed on both strands using the same primers employed for PCR at the DNA Sequencing and Synthesis Service of the Institute of Biochemistry and Biophysics, Polish Academy of Sciences, Warsaw, Poland. DNA sequences were assembled and edited using SeqMan (DNAStar, Lasergene, USA) and MegAlign (DNAStar, Lasergene, USA), with alignments to the published *A. phagocytophilum* 16S rRNA gene GU183908 and *B. burgdorferi* s.l. 16S rRNA gene DQ111061.

### Statistical analysis

Three multivariable logistic regression models were obtained according to the method described by Hosmer and Lemeshow (2000). A dog was considered as positive if it was PCR positive or serologically positive. Age, sex, origin, breed, tick control and region (voivodeship) were used as independent variables. The logistic regression analysis was performed using a nonautomatic selection of variables. Variables were added one by one (forward step) and the model refitted until *p* values were statistically significant (*p* < 0.05). The statistical analyses were performed using SPSS v15.0 software (SPSS Inc., Chicago, IL, USA).

## Results

While the highest prevalence of anti-*A. phagocytophilum* antibodies was observed in the serum of dogs from the *Lubelskie Voivodeship* (17.0 %), the highest prevalence of anti-*B. burgdorferi* and anti-*E. canis* antibodies was observed in the serum of dogs from the *Podlaskie* and *Mazowieckie Voivodeships* (18.0 and 4.0 % of the studied dogs, respectively) (Table 1). The concomitant occurrence of anti-*A. phagocytophilum* and anti-*B. burgdorferi* antibodies was observed in the serum samples of two dogs from the *Lubelskie Voivodeship* and two dogs from the *Mazowieckie Voivodeship*. The *D. immitis* antigen was detected in serum of none of the studied dogs.

**Table 1:** Univariate and multivariate analysis of *B. burgdorferi*, *A. phagocytophilum* and *E. canis* seropositivity/PCR infection in dogs from Eastern Poland

Variable	Category	No of dogs	Bb seroprevalence (%)/PCR prevalence (%)	Ap seroprevalence (%)/PCR prevalence (%)	Ec seroprevalence (%)/PCR prevalence (%)	Agent	OR	95 % CI	*p*
Origin of the animals	Country	243	19.7/8.3	14.6/1.9	0.6/1.9	Bb	3.66	1.80–7.44	<0.001***
	Town	157	5.3/2.9	3.7/7.0	2.1/2.1	Ap	2.94	1.41–6.14	0.004**
						Ec	–	–	0.505
Age	>1	115	14.4/5.3	9.1/4.2	2.1/1.8	Bb	–	–	0.052
	0–1	285	2.6/4.3	5.2/7.0	0.0/2.6	Ap	–	–	0.633
						Ec	–	–	0.332
Tick control	Yes	306	7.2/4.9	3.3/5.9	0.7/2.0	Bb	0.29	0.14–0.60	0.001**
	No	94	23.4/5.3	23.4/2.1	4.3/2.1	Ap	0.17	0.08–0.37	<0.001***
						Ec	–	–	0.095
Sex	Male	291	9.3 /5.2	8.2/3.4	0.7/0.3	Bb	–	–	0.699
	Female	109	15.6/4.6	7.3/9.2	3.7/6.4	Ap	–	–	0.479
						Ec	0.09	0.03–0.34	<0.001***
Breed	Pure	280	26.7/8.3	10.8/5.8	1.7/4.2	Bb	6.31	3.12–12.77	<0.001***
	Mixed	120	4.3/3.6	6.8/4.6	1.4/1.1	Ap	–	–	0.629
						Ec	–	–	0.276
Voivodship^1^	Lubelskie	100	9.0/3.0	17.0/8.0	1.0/2.0	Bb	R	R	R
						Ap	R	R	R
						Ec	R	R	R
	Mazowieckie	100	11.0/4.0	9.0/8.0	4.0/2.0	Bb	1.82	0.72–4.59	0.204
						Ap	0.61	0.28–1.30	0.196
						Ec	–	–	0.675
	Podkarpackie	100	6.0/3.0	3.0/2.0	1.0/2.0	Bb	0.50	0.16–1.61	0.249
						Ap	0.05	0.01–0.15	<0.001***
						Ec	–	–	0.913
	Podlaskie	100	18.0/10.0	3.0/2.0	0.0/2.0	Bb	3.07	1.20–7.82	0.019*
						Ap	0.08	0.03–0.26	<0.001***
						Ec	–	–	0.455

*p < 0.05

**p < 0.01

***p < 0.001

*R* Lubelskie was used as a reference category for multivariate analysis, – variable not included in the multivariate model, *Ap Anaplasma phagocytophilum*, *Bb Borrelia burgdorferi*, *Ec Ehrlichia canis*

The DNA of *A. phagocytophilum* was detected in the blood of six dogs (6 %) from the *Lubelskie Voivodeship* and five dogs (5 %) from the *Mazowieckie Voivodeship* by means of the PCR test. The amplified fragments of the 16S rRNA gene were 99.8–100.0 % homologous with the reference sequence GU183908. The SNAP 4Dx test did not reveal antibodies in any of the dogs, which might have indicated an early stage of infection, and no clinical signs were found of granulocytic anaplasmosis. At the same time, the PCR test revealed the DNA of *B. burgdorferi* in 20 dogs (*Lubelskie Voivodeship*, *n* = 3; *Mazowieckie Voivodeship*, *n* = 4; *Podkarpackie Voivodeship*, *n* = 3; *Podlaskie Voivodeship*, *n* = 10). The sequences of the PCR products showed 99.0 % similarity within the 16S rRNA partial sequence of *B. afzelii* (DQ111061). The results of the molecular tests were in line with the results of the serological tests in ten dogs (*Lubelskie Voivodeship*, *n* = 1; *Mazowieckie Voivodeship*, *n* = 1; *Podkarpackie Voivodeship*, *n* = 2; *Podlaskie Voivodeship*, *n* = 6). None of the dogs were shown to contain the DNA of *E. canis* in the blood during the PCR test.

The variables included in the regression logistic models for *B. burgdorferi*, *A. phagocytophilum* and *E. canis* are showed in Table 1.

Tick control was included in *B. burgdorferi* (OR = 0.29, CI = 0.14–0.60) and *A. phagocytophilum* (OR = 0.17, CI = 0.08–0.37) as a protective factor, while the origin (rural) was included as a risk factor for *B. burgdorferi* (OR = 3.66, CI = 1.80–7.44) and *A. phagocytophilum* (OR = 2.94, CI = 1.41–6.14) infection. In addition, the breed (pure) (OR = 6.31, CI = 3.12–12.77) was a risk factor only for *B. burgdorferi* infection, and sex (female) (OR = 0.09, CI = 0.03–0.34) was a risk factor only for *E. canis.* The region was also included as a risk factor in *B. burgdorferi* and *A. phagocytophilum* models, varying the Voivodeship with higher risk according to the agent.

## Discussion

The results of our own studies indicated that eastern Poland is at risk of CVBD. The most frequently observed antibodies are the antibodies against *Rickettsiae* of *A. phagocytophilum* and *Spirochaetes* of *B. afzelii.* These observations are reflected in practice. Among all canine transmissible diseases reported in this region of Europe, granulocytic anaplasmosis and Lyme disease are the most frequent causes of veterinary medical consultations, with the most common of these being babesiosis. Information on the prevalence and the factors affecting the distribution of babesiosis in dogs in Poland were presented extensively in an earlier paper (Adaszek et al. 2011), so in this article we focused on other CVBD listed in dogs in our country.

All studied dogs were free of *D. immitis* antigen. This is not surprising, since this parasite, as opposed to *D. repens*, is not endemic in Poland (Demiaszkiewicz et al. 2014). In addition, bearing in mind the fact that the animals used in the study did not travel beyond the Polish borders, they had practically no possibility of contact with these nematodes.

In Europe, *A. phagocytophilum* is transmitted by the tick *I. ricinus*, whose distribution range is limited to areas of high humidity and cold temperatures. Our results revealed a high seropositivity for this agent in eastern Poland (8.0 %). In prior studies conducted in dogs attending veterinary clinics in the eastern parts of Spain and in Finland, similar rates of 8.0 and 5.3 %, respectively, were reported (Miró et al. 2013; Pérez Vera et al. 2014). Despite the good sensitivity and specificity of the test (99.1 and 100.0 % for *A. phagocytophilum*, 92.0 and 100.0 % for *B. burgdorferi*, and 99.0 and 100.0 % for *E. canis*), serological cross-reactivity, e.g. between *A. phagocytophilum* and *A. platys*, has been described in experimentally infected dogs (Chandrashekar et al. 2010). Thus, PCR is required in order to identify the *Anaplasma* species. Our results could therefore indicate exposure to the *Anaplasma* genus with no information provided at the species level.

It should be emphasized, however, that so far no *A. platys* infections have been reported in dogs in Poland, whereas the occurrence of *A. phagocytophilum* has been regularly observed in the bodies of ticks and animals suffering from clinical granulocytic anaplasmosis (Adaszek et al. 2009; Adaszek et al. 2013; Dzięgiel et al. 2014). Our own studies revealed the occurrence of the DNA of these microorganisms in the blood of 11 dogs participating in the study. An early stage of infection might be possible, as the SNAP 4Dx ® test did not reveal anti-*A. phagocytophilum* antibodies in any of the dogs. On the other hand, the lack of DNA amplification of *A. phagocytophilum* from the dogs that were seropositive could be related to immunological elimination after infection, or a low level of infection, and therefore a low concentration of DNA in the blood sample (Miró et al. 2013).

Antibodies against *B. burgdorferi* were detected in 44 dogs (11.0 %). The results of the serological test were in line with the results of the molecular test in ten dogs. This confirms that the SNAP 4Dx ® kit may be used in the diagnosis of the acute as well as chronic stage of the infection.

The detection of the concomitant occurrence of anti-*A. phagocytophilum* and anti-*B. burgdorferi* antibodies in the serum samples from dogs suggests that both of the microorganisms may be concomitantly transmitted by ticks. Co-infections considerably hinder the diagnosis of the disease, making it severe and undermining the efficacy of the treatment. Even though, in the present work, such infections were observed only in four animals, their occurrence should be always considered in clinical practice, especially if we are dealing with cases of anaplasmosis or borreliosis resistant to therapy (Dzięgiel et al. 2014).

The bacterium *E. canis* is transmitted by *R. sanguineus*. This tick is not commonly found on dogs in Poland, which explains why the antibodies specific to these bacteria were found in only 1.5 % of the studied dogs, with the PCR test results for *E. canis* showing negative in all dogs participating in the study. Similarly, a low seroprevalence for *E. canis* (0.26 %) in dogs in Poland was demonstrated by Krämer et al. (2014).

The analysis of results presented above could suggest that the serological results shown here for *E. canis* could be only false-positive results and *E. canis* was not prevalent in Poland. Confirmation of this may be the lack of description of clinical cases of canine monocytic ehrlichiosis in Poland in the available literature.

Our own studies have also shown that some factors may favour the occurrence of CVBD in dogs. As shown by the results of statistical analysis, the conditions in which dogs are kept can affect the development of CVBD, and our own study shows that the *A. phagocytophilum* and *B. burgdorferi* infections were observed more frequently in animals from rural areas (*p* = 0.004 for *A. phagocytophilum* and *p* < 0.001 for *B. burgdorferi*). This may be connected with the greater exposure to arachnids in this group of dogs and possibly with cruder living conditions than typically found with city dogs. Similar observations have also been made in the case of other vector-borne diseases in dogs (Welc-Falęciak et al. 2009).

Age has not been shown to favour the occurrence of the infection. Sex was a risk factor only for *E. canis* infection (*p* < 0.001).

The situation with breed predispositions is similar. We observed higher infection levels for *B. burgdorferi*, more often in pure-breed dogs than in mixed breed dogs. Also the results of a statistical analysis have confirmed that pure-breed dogs are more prone to Lyme disease (*p* < 0.001). However, in practice, no significant predispositions were observed to indicate that one breed may be more prone to infection than another. It might be expected that, due to their more frequent exposure to ticks, hunting dogs and possibly shepherd dogs may be at greater risk of anaplasmosis, borreliosis and ehrlichiosis. However, these diseases are not connected with the predispositions of a particular breed but rather with living conditions and the nature of their work.

The use of preventive measures against ectoparasites significantly limits the development of *B. burgdorferi* (*p* = 0.001) and *A. phagocytophilum* (*p* < 0.001) infections. This indicates that regularly administering acaricides is an effective measure for these infections.

Several other factors may also have led to the observed emergence of vector-borne diseases, such as climate change, improvements in available diagnostic techniques, development of commercial serological screening tests and increased awareness among veterinarians and owners of diseases transmitted by arthropods (Beugnet and Marie 2009).

In conclusion, there still remains a need for research targeted at the diagnosis, treatment and prevention of CBVD. Information on the prevalence and geographical distribution of these infections is essential for effective planning of control measures and their surveillance thereafter. This preliminary overview of the current situation in eastern Poland requires further work to complete the prevalence map of agents causing CBVD in Poland.
